# Integrated Signals of Jasmonates, Sugars, Cytokinins and Auxin Influence the Initial Growth of the Second Buds of Chrysanthemum after Decapitation

**DOI:** 10.3390/biology10050440

**Published:** 2021-05-16

**Authors:** Daojin Sun, Luyao Zhang, Qi Yu, Jiali Zhang, Peiling Li, Yu Zhang, Xiaojuan Xing, Lian Ding, Weimin Fang, Fadi Chen, Aiping Song

**Affiliations:** 1 State Key Laboratory of Crop Genetics and Germplasm Enhancement, Key Laboratory of Landscaping, Ministry of Agriculture and Rural Affairs, College of Horticulture, Nanjing Agricultural University, Nanjing 210095, China; daojinsun@163.com (D.S.); 2019804185@njau.edu.cn (L.Z.); 2019104103@njau.edu.cn (Q.Y.); 2020804199@stu.njau.edu.cn (J.Z.); yuzhang0315@njau.edu.cn (Y.Z.); xingxiaojuan@njau.edu.cn (X.X.); dinglian@njau.edu.cn (L.D.); fangwm@njau.edu.cn (W.F.); chenfd@njau.edu.cn (F.C.); 2 Henan Key Laboratory of Tea Comprehensive Utilization in South Henan, Xinyang Agriculture and Forestry University, Xinyang 464000, China; lipl@xyafu.edu.cn

**Keywords:** decapitation, third-generation transcriptome, phytohormone, shoot branching, *Chrysanthemum morifolium*

## Abstract

**Simple Summary:**

We sequenced the first third-generation transcriptome of chrysanthemum and analyzed the factors involved in the outgrowth of the second buds after decapitation. In addition to classic hormones (auxin, cytokinins, and strigolactone), jasmonates and sugars were also found to be involved in this process, which might be related to the initiation of dormant buds.

**Abstract:**

Decapitation is common in horticulture for altering plant architecture. The decapitation of chrysanthemum plants breaks apical dominance and leads to more flowers on lateral branches, resulting in landscape flowers with good coverage. We performed both third- and second-generation transcriptome sequencing of the second buds of chrysanthemum. This third-generation transcriptome is the first sequenced third-generation transcriptome of chrysanthemum, revealing alternative splicing events, lncRNAs, and transcription factors. Aside from the classic hormones, the expression of jasmonate-related genes changed because of this process. Sugars also played an important role in this process, with upregulated expression of sucrose transport-related and *TPS* genes. We constructed a model of the initial growth of the second buds after decapitation. Auxin export and sugar influx activated the growth of these buds, while the JA-Ile caused by wounding inhibited the expression of *CycD* genes from 0 h to 6 h. After wound recovery, cytokinins accumulated in the second buds and might have induced *ARR12* expression to upregulate *CycD* gene expression from 6 h to 48 h, together with sugars. Therefore, jasmonates, cytokinins, sugars, and auxin work together to determine the fate of the buds of plants with short internodes, such as chrysanthemum.

## 1. Introduction

Control of plant architecture is one of the most sought-after objectives of breeders, with the green revolution having provided enormous economic value since the 1960s–1970s [1,2]. The number, length, and angle of the lateral branches of plants determine plant architecture [3]. Shoot branching involves multiple complex biological processes that can be divided into two main processes: bud initiation and bud outgrowth [3]. The axillary meristem arises in the axils of leaves along the primary shoot axis and then produces several leaves that form buds. These buds can develop into vegetative branches or can remain dormant. All buds have the potential to grow into branches, but not all buds have the opportunity to grow and develop, due to the limited resources provided for the plant lifecycle, so plant architecture is mostly regulated by the process of bud outgrowth [4]. An improved understanding of the molecular mechanisms underlying branching provides a foundation for breeding new cultivars with expected performance and output. Endogenous, developmental, and environmental factors work together to determine the fate of buds; specifically, whether to break dormancy or not [5].

Thimann and Skoog demonstrated that the removal of the first shoot of broad bean plants promoted the growth of lateral buds, and this growth was largely inhibited by the application of exogenous auxin to the stump of decapitated plants [6,7]. The removal of the first bud is called ‘decapitation’, which is commonly applied in horticultural production. Auxin is synthesized in both younger buds and leaves and is transported basipetally through the polar auxin transport stream (PATS) to inhibit the growth of lateral buds, which is referred to as apical dominance. However, previous studies have indicated that auxin does not enter buds and instead functions indirectly [5,8,9]. There are two models that support the indirect role of auxin: the second messenger model and auxin canalization model. In the second messenger model, auxin regulates the biosynthesis of cytokinins (CKs) and strigolactone (SL) [5]. Auxin can suppress the expression of the rate-limiting CKs biosynthesis-related gene *IPT*, and the CKs content was shown to increase with decreasing indoleacetic acid (IAA) levels after decapitation [10,11,12]. In contrast, auxin upregulates the expression of *MAX3*/*CCD7* and *MAX4*/*CCD8*, which encode plastidic carotenoid cleavage dioxygenases [13,14]. The auxin canalization system formed in the place where the auxin flux was canalized. Depending on these positive feedback loops, auxin enhanced its polar transport from the leaves to roots to form vascular strands [15]. It is beneficial for the growth of buds to export auxin to PATS and form canalized auxin transport. The dominant first bud inhibited the growth of lateral buds by exporting more auxin to PATS in the top. Therefore, the apical bud and lateral buds compete for access to exported auxin [16]. Once decapitated, lateral buds can export more auxin than before and resume their growth [5].

In contrast to auxin, CKs directly promote the growth of buds, and the three main active forms of CKs are isopentenyladenine (iP), zeatin (ZT), and dihydrozeatin (DZ) [17]. CKs are mainly synthesized in the tips of roots and shoots [11,18] and can move acropetally to exert their effects. Exogenous CKs can induce the activation of the cell cycle-related genes involved in the signal transduction pathways to activate dormant buds [19,20].

Since the discovery of SL in 2008 [21,22], this hormone has been found to be involved in different biological processes, including shoot branching. Exogenous application of SL inhibited the growth of buds, and many SL synthesis or signaling mutants display bushy phenotypes [23]. SL binds to the D14 protein and activates it by inducing a major conformational change [24], which facilitates the binding of D14 and MAX2/D3, leading to the ubiquitination and degradation of D53 proteins via the 26S proteasome [25,26]. SL has also been shown to act as an upstream component of auxin by promoting the depletion of PIN1 from the plasma membrane, which inhibits the export of auxin in axillary buds and reduces their ability to grow [27].

Jasmonates (JAs) mainly consist of jasmonic acid (JA), its methyl ester methyl jasmonate (MeJA), and its isoleucine conjugate (JA-Ile), all of which are derivatives of a class of fatty acids. JAs are broadly involved in plant resistance-related pathways, the response to external damage (mechanical, herbivore, and insect damage), and other biotic or abiotic stresses [28]. As in the auxin signal transduction model, JA-Ile binds to JAZ and COI1 proteins, leading to the degradation of JAZs and inducing the expression of downstream genes.

Sugars have been recently considered as new regulators of shoot branching. Sugars play a pivotal role in the primary metabolism of plants and are needed as carbon sources, as energy sources, and for cell wall synthesis [29]. In addition to those roles, sugars play a signaling role in shoot branching in the initial stage of shoot outgrowth [23]. This process occurs prior to hormone signal transduction because sucrose can travel at a faster speed (150 cm/h) than auxin [30]. Apical dominance can also be attributed to a relatively strong sugar sink of the shoot tip, which deprives lateral buds of sugars [31,32].

*Chrysanthemum morifolium* is a traditional Chinese flower that occupies an important position in both the cut flower and potted flower markets. Producers control the branching of chrysanthemum plants by decapitating or removing lateral buds manually, which constitutes nearly 1/3 of the cost [33]. Therefore, it is important to understand the mechanisms of shoot branching for breeding new cultivars with ideal branching characteristics. Transcriptome sequencing is a powerful tool for gene discovery and quantification. We chose six different stages (0 h, 3 h, 6 h, 12 h, 24 h, and 48 h) after decapitation and sampled the second buds at each time point.

Large-scale transcriptomes are usually obtained by next-generation sequencing (NGS). However, NGS is unable to identify transcript features such as new splice sites, transcription start sites, and poly(A) sites, due to the short lengths [34], and fails to connect two alternative splices 1000 bp apart [35]. The emergence of third-generation sequencing (TGS) has helped researchers obtain full-length sequences, which are essential for accurate transcript reconstruction and gene annotation [36]. Due to the extensive variance, high heterozygosity, and polyploidy, it has been difficult to obtain a *de novo* whole-genome assembly of *Chrysanthemum morifolium* [37]. Thus, to construct a reference genome set, we sequenced a mixed sample of second buds of chrysanthemum plants after decapitation at each time point via the ISO-Seq platform, the data of which constitutes the first TGS transcriptome of chrysanthemum plants. In addition, we sequenced the NGS transcriptomes of the same second buds on an RNA-Seq platform. In the present study, we analyzed the features of the TGS transcriptome, the expression profiles of the genes, and the contents of hormones and proposed a model of the initial growth of the second buds of chrysanthemum after decapitation.

## 2. Materials and Methods

### 2.1. Plant Materials, Tissue Preparation, and RNA Extraction

Seedlings of the *Chrysanthemum morifolium* variety ‘Jinba’ were cut and cultivated in a greenhouse at Nanjing Agricultural University (Nanjing, China), with a day/night temperature of 26/18 °C and ~70% relative humidity. When the seedlings had 20 leaves, we removed the apical bud and two leaves around it for each seedling, and we collected samples of the second buds at various time points (0 h, 3 h, 6 h, 12 h, 24 h, and 48 h). The samples were frozen in liquid nitrogen immediately and stored at −80 °C. Total RNA was extracted using a commercial RNA isolation kit (Huayueyang, Beijing, China), according to the provided protocol. Six cells from each time point were mixed for single molecular real-time (SMRT) sequencing, and eighteen samples of the six time points were subjected to Illumina sequencing.

### 2.2. Measurements of Endogenous Plant Hormones

The quantification of plant hormones was performed as previously described, with slight modifications [38]. Since the measurement of the plant hormones required a greater bud size than the sequencing, we collected the second, third, and fourth buds from the top to bottom of each plant at each time point. The samples at each time point were biologically replicated three times, and each biological replicate weighed at least 0.6 g. After sampling, we froze the collected buds in liquid nitrogen, which were subsequently ground into powder and extracted with methanol/water/formic acid (15:4:1, V/V/V). After being evaporated to dryness in a gas stream of nitrogen, the combined extracts were reconstituted in 80% methanol (V/V) and filtered (polytetrafluoroethylene, 0.22 mm; Anpel). Then, the content of auxin, CKs, SL, and JAs was detected using the AB 6500+ QTRAP^®^ LC-MS/MS platform, which was analyzed by MetWare Biotechnology (http://www.metware.cn/, accessed on 2 April 2021).

### 2.3. PacBio cDNA Library Construction and SMRT Sequencing

Full-length cDNAs were synthesized using a SMARTer™ PCR cDNA Synthesis Kit (Takara Clontech Biotech, Dalian, China) with mRNA templates. The cDNAs were fractionated and selected using a BluePippin^TM^ Size Selection System (Sage Science, Beverly, MA, USA), resulting in cDNA libraries of different-sized fragments (1–2 k, 2–3 k, and 3–6 k). cDNA libraries were then generated using a Pacific Biosciences DNA Template Prep Kit 2.0 (Menlo Park, CA, USA) and sequenced on a PacBio RS II platform (Menlo Park, CA, USA). Nonchimeric full-length sequences were identified from reads of inserts (ROIs) on the basis of the presence of poly(A) tail signals, 5′ adapter sequences, and 3′ adapter sequences. Then full-length transcripts were clustered into consensus transcripts according to their sequence similarity, using iterative isoform-clustering. Non-full-length transcripts were also clustered and all consensus transcripts were polished using Quiver software (http://www.pacbiodevnet.com/quiver/, accessed on 2 April 2021) [39]. Low-quality transcript sequences identified by Quiver were corrected by Illumina transcriptome data. Finally, high-quality sequences and low-quality sequences were removed redundancy using CD-HIT [40].

### 2.4. Illumina cDNA Library Construction and Sequencing

Illumina cDNA libraries were generated using a NEBNext Ultra RNA Library Prep Kit (NEB, Ipswich, MA, USA) following the standard protocol. The libraries were sequenced on Illumina HiSeq X Ten platform (San Diego, CA, USA). Both the PacBio and Illumina transcriptome sequencing were conducted by Beijing Biomarker (Beijing, China).

### 2.5. Prediction of Alternative Splicing and lncRNAs and Annotation of Transcription Factors (TFs)

On the basis of the IsoSeq_AS_de_novo [41], we identified possible alternative splicing events. The identification of lncRNAs was performed by four protein-coding assessment software programs: coding potential calculator (CPC) [42], coding-noncoding index (CNCI) [43], coding potential assessment tool (CPAT) [44], and the Pfam database [45]. The target mRNAs of lncRNAs were predicted using LncTar [46], while the annotation of TFs was performed using iTAK [47].

### 2.6. Functional Annotation and Identification of Differently Expressed Genes (DEGs)

Reads from the Illumina sequencing were mapped to SMRT cDNA libraries by Bowtie2 [48] and quantified with the fragments per kilobase of transcript per million mapped reads (FPKM) method via RSEM [46]. Functional annotations were performed with the NCBI-Nr (https://www.ncbi.nlm.nih.gov/refseq/about/nonredundantproteins/, accessed on 2 April 2021), SwissProt (www.expasy.ch/sprot, accessed on 2 April 2021), Gene Ontology (GO) (http://geneontology.org/, accessed on 2 April 2021), COG (www.ncbi.nlm.nih.gov/research/cog-project/, accessed on 2 April 2021), KOG (www.hsls.pitt.edu/obrc/, accessed on 2 April 2021), Pfam (http://pfam.xfam.org/, accessed on 2 April 2021), and Kyoto Encyclopedia of Genes and Genomes (KEGG) (www.kegg.jp/, accessed on 2 April 2021) databases by BLASTX alignment [49]. DEGs between comparison groups were identified by DESeq2 [50], and heatmaps were constructed with the R package pheatmap.

## 3. Results

### 3.1. Sequence Samples of Different Time Points via ISO-Seq and RNA-Seq

To understand the expression profiles in the lateral buds in depth, the RNA of a sample (F01) was sequenced using ISO-Seq on a PacBio platform with six SMRT cells. A total of 297,519 ROIs were generated, and 43.49% (129,388) were identified as full-length nonchimeric (FLNC) reads on the basis of the presence of poly(A) tail signals, 5′ adapter sequences, and 3′ adapter sequences. TGS has the advantage of generating long reads (average 4–8 kb), but it also succumbs to a relatively high error rate (up to 15%), which can be corrected by self-correction via circular consensus reads and NGS data. In addition, RNA-Seq was used to quantify gene expression levels. A total of 18 samples were sequenced using RNA-Seq, and the samples at each stage were replicated three times: 0 h (T01, T02, and T03), 3 h (T04, T06, and T19), 6 h (T07, T08, and T09), 12 h (T10, T11, and T12), 24 h (T13, T14, and T15), and 48 h (T16, T17, and T18). A total of 424,681,517 clean reads, with a total length of 126.91 Gb, were generated on the Illumina platform, and the Q30 values of all the samples were larger than 90% (Table 1). All FLNC sequences were then clustered by SMRT Analysis (v2.3.0), generating 68,355 consensus transcripts. After removing redundancy by CD-HIT [40], 62,117 nonredundant full-length sequences were ultimately generated in the F01 libraries. Based on the length of different sequences, the 62,117 sequences were separated into three libraries, the statistical data of which are shown in Table 2.

### 3.2. Features of the TGS Transcriptome and Functional Annotations

Through alternative splicing, pre-mRNAs can be spliced into different transcripts with different exons, which contributes to transcriptome diversity, the coding capacity of genomes, and gene regulatory mechanisms in eukaryotes [51]. The full-length cDNA library of ISO-Seq allows the identification of alternative splicing events in our transcriptomic data. In the present study, 1415 alternative splicing events were predicted in the full-length cDNA library (Table S1). As there is no published reference genome for *Chrysanthemum morifolium*, the types of alternative splicing could not be identified.

lncRNAs are transcripts longer than 200 nt without protein-coding capacity and that play a regulatory role in eukaryotes. We adopted four frequently used methods (CPC, CNCI, Pfam, and CPAT) to predict lncRNAs in the F01 transcript library. Using these four prediction software programs and taking the transcript identified by all four programs, we identified 2738 lncRNA transcripts (4.08% of all transcripts) (Figure S1a, Table S2) and predicted their possible target transcripts (Table S3).

Afterward, TFs were identified in the F01 library. There were 2835 TFs (4.56% of all transcripts) annotated in the F01 transcript libraries, and the 10 most common types were MYB, C3H, RWP-RK, B3, bHLH, GRAS, HB, WRKY, AP2/ERF, and C2H2 TFs (Figure S1b).

To better understand the biological processes after decapitation, eight databases (GO, KEGG, KOG, Pfam, SwissProt, COG, eggNOG, and Nr) were used to functionally annotate SMRT transcripts (Table S4). With the exception of the 2748 lncRNAs, 57,972 transcripts (97.63% of all transcripts) were annotated, and the remaining 1407 transcripts had no hits in any databases (Table 3).

### 3.3. GO and KEGG Annotation of DEGs

Genes in different treatment comparisons and whose expression significantly changed (false discovery rate [FDR] ≤ 0.01, |log2| ≥ 1) were identified as DEGs. Pairwise comparisons for all time points were performed (Table 4). The two most significant comparisons were ‘0 h vs. 6 h’ and ‘6 h vs. 48 h’, in which there were 5247 and 4377 DEGs, respectively, indicating that 6 h and 48 h were the two pivotal time points after decapitation. Therefore, we used the GO and KEGG databases to annotate the DEGs in these two groups. Interestingly, the results of both GO and KEGG analysis were nearly identical in the ‘0 h vs. 6 h’ and ‘6 h vs. 48 h’ comparisons, which showed similar and continuously changing processes in the buds. The GO annotations were classified into three groups: biological process (BP), cellular component (CC), and molecular function (MF). The BP category was mostly enriched in ‘metabolic process’, ‘cellular process’, and ‘single-organism process’. In the CC category, ‘cell’, ‘cell part’, and ‘organelle’ were the most predominant, and most genes annotated to MF were ‘catalytic activity’ and ‘binding’ (Figure S2a,b). These results suggested that there were various processes associated with metabolic activity, cellular activity, and biological signal transduction after decapitation.

**Figure 1:**
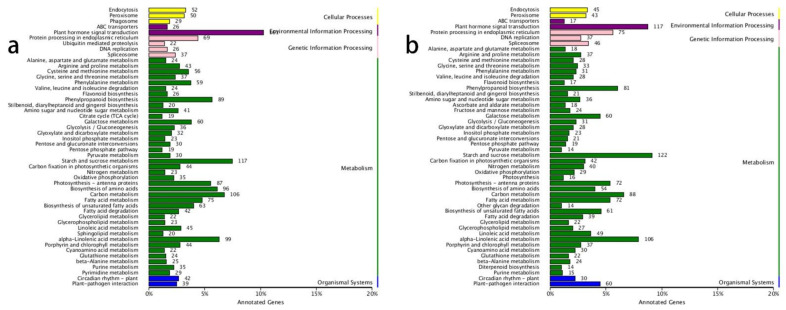
KEGG annotations in the ‘0 h vs. 6 h’ and ‘6 h vs. 48 h’ comparisons. (**a**) KEGG annotations in the ‘0 h vs. 6 h’ comparison; (**b**) KEGG annotations in the ‘6 h vs. 48 h’ comparisons.

The KEGG annotation results revealed that the most enriched gene pathways in both the ‘0 h vs. 6 h’ and ‘6 h vs. 48 h’ comparisons were ‘plant hormone signal transduction’, ‘starch and sucrose metabolism’, ‘alpha-linolenic acid metabolism’, and ‘carbon metabolism’ (Figure 1a,b). This indicated that the plant hormones played vital roles in lateral shoot growth and that carbon-related metabolic processes accelerated after decapitation, which correlated with the growth of lateral buds.

### 3.4. DEGs Related to Hormones

After decapitation, the dormant shoots became active and started to grow. For a more in-depth understanding, we analyzed the DEGs related to hormones based on KEGG annotations. Due to the high polyploidy of cultivated chrysanthemums, one gene may present several copies and transcripts in transcriptomes.

**Figure 2:**
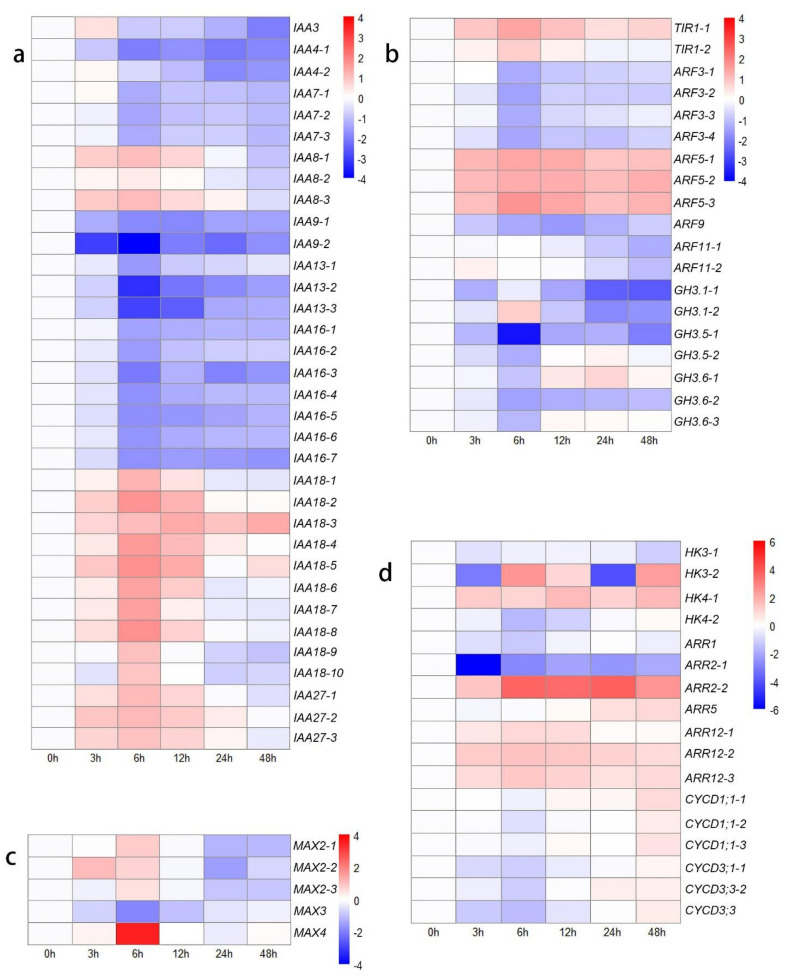
Heat map showing log2-normalized expression fold changes at each time point for hormone-related genes after decapitation. (**a**) *IAA* family genes; (**b**) *TIR*, *ARF,* and *GH3* family genes; (**c**) SL-related genes; (**d**) CKs-related genes.

In the present study, DEGs associated with *IAA* and *ARF* genes exhibited various expression trends on the basis of different types (Figure 2a,b). *IAA4*, *IAA7*, *IAA9*, *IAA13,* and *IAA16* were expressed at lower levels after decapitation than at 0 h. The expression of *IAA3*, *IAA8*, *IAA27,* and six transcripts of *IAA18* was first upregulated from 0 h to 12 h or 24 h but then downregulated at 48 h. The expression of only four transcripts of *IAA18* was upregulated from 0 h to 48 h compared to their expression at 0 h. The expression of two transcripts of *TIR1* was upregulated, and four DEGs encoding *ARF5* were highly expressed after decapitation, while the expression of the other seven DEGs encoding *ARF3*, *ARF9,* and *ARF11* was downregulated (Figure 2b). These findings may be due to the decreased levels of auxin, as the *GH3* gene family members exhibited relatively low expression levels.

In the SL-related signal transduction, *MAX4* and three transcripts of *MAX2* were highly expressed at 6 h compared with 0 h, after which their expression decreased. The expression of *MAX3* was downregulated from 0 h to 48 h (Figure 2c). These results indicated that the decreased IAA might decrease the biosynthesis of SL. In CKs-related signal transduction, *histidine kinase* (*HK*), *two component response regulator ARR-B* (*B-ARR*), a *two-component response regulator ARR-A* family member (*A-ARR*), and several cell cycle-related genes were identified among the DEGs in different comparisons (Figure 2d). In the ‘0 h vs. 48 h’ comparison, three transcripts of *HK* (*HK3-1*: downregulated, 2.23-fold change; *HK3-2*: upregulated, 5.06-fold change; *HK4*: upregulated, 1.14- to 3.20-fold change), one transcript of *A-ARR* (*ARR5*: upregulated, 1.90-fold change), and four transcripts of *B-ARR* (*ARR2-1*: downregulated, 4.05-fold change; *ARR2-2*: upregulated, 5.78-fold change; *ARR12*: upregulated, 1.12- to 1.90-fold change) were found. In terms of cell cycle-related genes, the expression of six *D-type cyclin* (*CycD*) genes was first downregulated (1.31- to 3.04-fold change) in the ‘0 h vs. 6 h’ comparison group and then upregulated (2.18- to 4.23-fold change) in the ‘6 h vs. 48 h’ comparison group (Figure 2d). These results indicated that the outgrowth of lateral buds was first blocked from 0 h to 12 h but then upregulated to a higher rate at 48 h compared to at 0 h.

**Figure 3:**
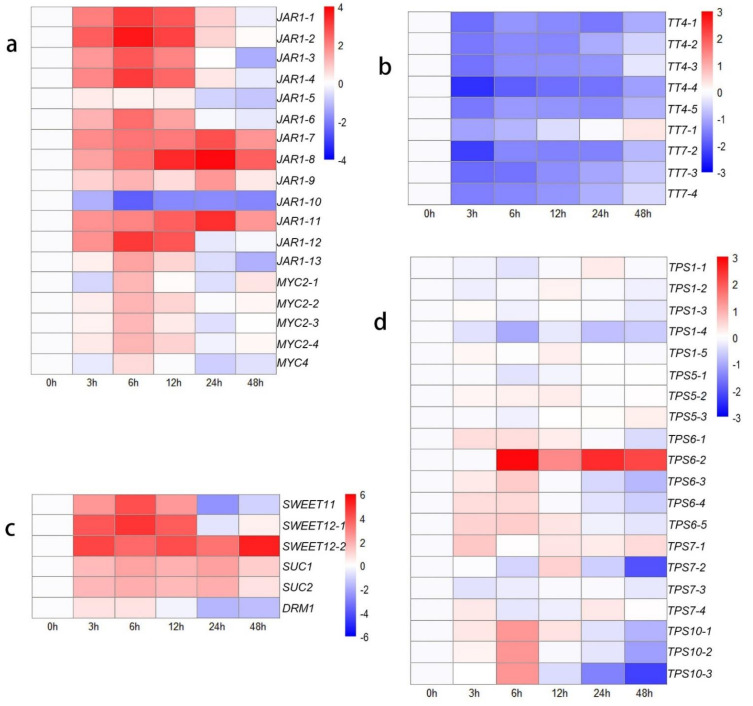
Heat map showing log2-normalized expression fold changes at each time point for JA-related genes and metabolism-related genes after decapitation. (**a**) JA-related genes; (**b**) flavonoid-related genes; (**c**) sucrose transport-related genes; (**d**) *TPS* family genes.

To clarify these interesting results, the other genes in the ‘plant hormone signal transduction’ KEGG pathway were identified, which indicated that many genes are involved in JA signal transduction (Figure 3a). The genes that respond to JAs showed high activity after decapitation, which may be attributed to the wound response caused by this phenomenon. JA-Ile is the main active form in the JA response pathway; the conjugation of JA and Ile is catalyzed by *jasmonate resistant 1* (*JAR1*) [52]. The expression levels of most transcripts of *JAR1* increased from 0 h to 6 h (upregulated: 2.33- to 12.6-fold change), suggesting accelerated JA-related reactions in plants. *MYC2* and *MYC4* are the key downstream TFs involved in JA signaling and participate in the wound response [53,54]. The expression of six *CycD* genes was downregulated at the initial time. This result may be attributed to the wound response caused by mechanical decapitation treatment, so the transcription of cell cycle-related genes was blocked from 0 h to 12 h after decapitation. However, the growth state of the lateral buds was adjusted quickly, and the expression of cell cycle-related genes increased to a higher level, with increased CKs levels from 24 to 48 h compared to 0 h.

### 3.5. Analysis of Hormone Contents in Buds after Decapitation

**Figure 4:**
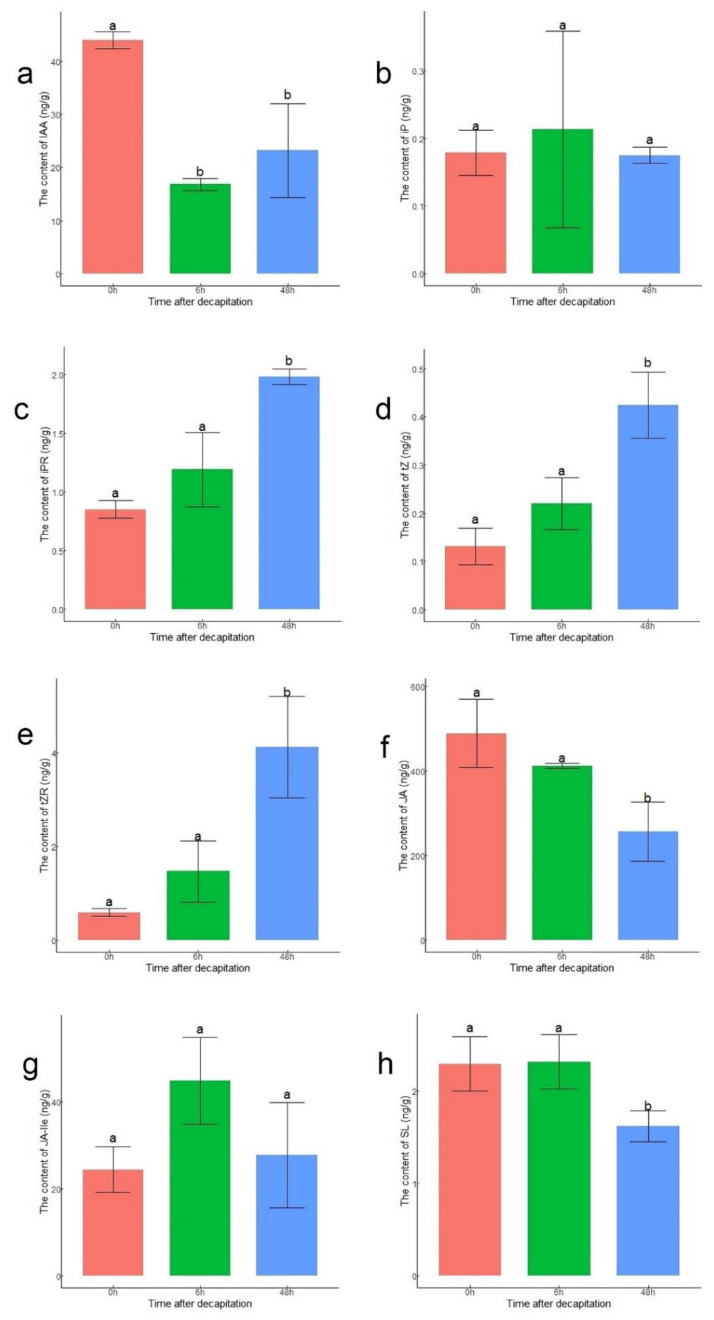
Contents of hormones in lateral buds at 0 h, 6 h, and 48 h after decapitation. (**a**) IAA; (**b**) iP; (**c**) iPR; (**d**) tZ; (**e**) tZR; (**f**) JA; (**g**) JA-Ile; (**h**) SL. The statistical analysis was performed via ANOVA with R version 4.0.3. The letters ‘a’ and ‘b’ above the bar represent significance at *p* < 0.05.

To verify the accuracy of the expression levels of DEGs related to hormones, the endogenous contents of IAA, CKs, SL, and JAs in lateral buds were measured at 0 h, 6 h, and 48 h after decapitation (Figure 4). The content of IAA at 6 h and 48 h decreased compared to that at 0 h (Figure 4a). Four active forms of CKs were measured. Except for those of iP, the contents of N6-isopentenyladenosine (iPR), trans-zeatin (tZ), and trans-zeatin riboside (tZR) were significantly higher at 48 h than at 0 h (Figure 4b–e). The JA content at 0 h and 6 h was significantly higher than that at 48 h. This might have been because the content of JA increased rapidly after decapitation (Figure 4f). Although there was no significant difference in content of JA-Ile among the three points, the content of JA-Ile was still highest at 6 h, corresponding to the upregulation of *JAR1* (Figure 4g). The content of SL was significantly lower at 48 h than at 0 h and 6 h (Figure 4h).

### 3.6. DEGs Related to Metabolic Activity

The KEGG annotation results revealed many genes annotated to the ‘starch and sucrose metabolism’ and ‘carbon metabolism’ pathways, indicating activated carbohydrate metabolism and energy transformation processes were occurring in the lateral buds at those time points. Therefore, we identified several sugar-related pathways to study the effect of sugars on bud outgrowth (Figure 3c,d). The SUC and SWEET sucrose transporters were expressed at higher levels from 3 h to 48 h than at 0 h. The expression of *DRM1*, a bud dormancy-related marker gene that is also a low-specificity sensor of sugars [55], decreased. The expression levels of *TPS1*, *TPS5,* and *TPS7* did not significantly change. Lastly, *TPS6* and *TPS10* transcript levels increased at 6 h, demonstrating that trehalose 6-phosphate (Tre6P) levels might increase.

There were many DEGs associated with the flavonoid pathway and phenylpropanoid pathway according to the KEGG annotation results, indicating that the phenylpropanoid and flavonoid derivatives actively changed after decapitation. We extracted the sequences, identified those genes, and constructed a heatmap showing their expression-change trends (Figure 3b). The results showed that the expression of the *TT4* and *TT7* genes was downregulated. *TT4* (*CHS*), encoding a chalcone synthase, synthesizes naringenin, and the *TT7* product synthesizes quercetin and kaempferol downstream of flavonoid biosynthesis. These results indicated that the synthesis of flavonoids, including naringenin, kaempferol, and quercetin, decreased.

## 4. Discussion

During the past few decades, three different plant hormones, namely auxins, CKs, andSL, have been recognized as key signals involved in bud outgrowth [4]. The synergistic and antagonistic relationships among these three hormones determine the fate of buds: dormancy or outgrowth. Recently, sugars have been found to be involved in several signal transduction pathways and to interact with the other regulatory networks involved in plant development [56,57]. Sugars are redistributed to lateral buds after decapitation, and exogenous application of sucrose promotes the growth of axillary buds [30]. This suggests that sugars act as important regulators in the shoot branching of plants.

In auxin signal transduction, ARFs rapidly activate the transcription of early response genes, such as members of the *Aux*/*IAA*, *SAUR*, and *GH3* gene families [58]. At low auxin levels, IAA/AUX proteins complex with ARFs and interfere with their transcriptional activity. However, at high auxin levels, TIR1/AFB proteins bind auxin and then act as F-box ubiquitin ligases to mediate the ubiquitination of Aux/IAAs, which relieves the repression of ARFs to activate or repress downstream gene expression [59]. *ARF* and *IAA* are functionally redundant, especially in polyploid plants. However, there were different expression patterns for both *ARF* and *IAA* family genes after decapitation, indicating functional diversification within *Chrysanthemum morifolium*. After the *ARF*s were annotated, the results showed that only the expression levels of *ARF5* increased, which was also the only positively acting ARF among the four ARFs in this study [60]. The expression levels of *IAA8* and *IAA18* were upregulated at the initial time point. Overexpression of *MP*/*ARF5* was shown to promote shoot regeneration, while the *arf5* mutant had a defect in shoot formation [61]. Therefore, *ARF5* in chrysanthemum might activate some cell cycle-related genes downstream and then promote shoot growth after decapitation. Compared with its wild type, the *Arabidopsis thaliana* gain-of-function *IAA8* mutant presented more lateral branches, short primary inflorescence stems, and decreased shoot apical dominance [62]. Moreover, the gain-of-function *IAA18* mutant *crane-2* exhibited a dwarf phenotype [63]. We propose that *ARF5* might interact with *IAA8* and *IAA18* to regulate the plant development processes of chrysanthemum plants after decapitation, indicating that auxin signal transduction is involved. The auxin levels in lateral buds decreased after decapitation, and the expression levels of *GH3.1*, *GH3.5,* and *GH3.6* also decreased. *GH3.1*, *GH3.5,* and *GH3.6* regulate auxin homeostasis by catalyzing the conjugation of IAA and amino acids, resulting in the degradation of auxin [64,65]. The downregulation of these three genes was consistent with the decreased content of IAA in the lateral buds.

Depending on hormone analysis, the lateral buds were able to export more IAA after the removal of the first bud, which is consistent with the auxin canalization model. Flavonoids, including quercetin, kaempferol, apigenin, and other aglycone molecules, have been shown to reduce the transport of IAA in PATS [66]. The mechanisms of these flavonoids have been predicted: one is that flavonoids might inhibit the activity of PGP transporters, and the other is that flavonoids might modulate the trafficking of cellular auxin-transport proteins [67]. Among the DEGs related to secondary metabolism, the expression levels of *TT4* and *TT7* decreased, indicating decreased cellular flavonoids in the buds. Other studies also found that flavonoids are associated with bud dormancy in plant species such as leafy spurge [68,69], grapevine [70], and rice [71]. Therefore, decapitation might lead to decreased levels of flavonoids in the lateral buds, which increases the export of IAA. The decreased content of flavonoids might be related to the breaking of bud dormancy in chrysanthemum plants.

Sugars play an essential role in bud outgrowth because they are both the source of nutrients and a component of signal transduction. In contrast to auxin, buds compete to absorb more sugars to grow into branches. Decapitation induced an increase in sucrose content in the lateral buds [30]. Sucrose from the source leaves is transported through the phloem to sink organs, and phloem unloading of sucrose depends on SUC and SWEET sucrose efflux carriers [72,73]. In *Chrysanthemum morifolium*, application of sucrose has been shown to induce increased expression levels of sugar transporter-encoding genes, and *SWEET*-overexpressing plants display elongated buds [74]. The upregulated expression of genes encoding sucrose transporters in the present study indicated that more sucrose was transported to the lateral buds after decapitation, which can be attributed to the removal of a strong sugar sink. After the decapitation of garden pea plants, Tre6P levels rose rapidly, together with increased efflux of sucrose from the phloem to the buds; thus, Tre6P acts as a signal of sucrose availability to initiate the growth of buds [75]. Tre6P has been identified as a signal for sucrose-related metabolites to modulate bud growth. Tre6P is synthesized by members of the *TPS* family, and overexpression of *TPP* in axillary buds delays the outgrowth of buds [76]. In the present study, the expression of *TPS6* and *TPS10* was upregulated at 6 h, which suggests a possible increased level of Tre6P. Therefore, the upregulation of sucrose carrier-encoding genes and *TPS* genes might activate the initial outgrowth of lateral buds in chrysanthemum plants within 6 h.

After decapitation, the second buds of chrysanthemum plants responded to wounding. The results of hormone analysis also indicated that JA-Ile was the active form of JA functioning in the wound response. JA-Ile played an important role in this stage, which might be related to the downregulation of *CycD* gene expression. Application of MeJA to cell suspension cultures of *Arabidopsis thaliana* inhibited cell growth and halted cell cycle progression in the G2 phase [77]. In *Taxus cuspidata* suspension cultures, MeJA impeded the G1/S transition phase, resulting in an increased number of G0/G1 phase cells and a decrease in all dividing cells [78]. The duration of the wound response continued from 0 h to 12 h, after which the expression of the cell cycle-related genes was induced again. The *CycD* gene expression returned to relatively high levels from 24 h to 48 h, indicating the recovery of the buds. The CKs content also increased significantly, but the expression levels of *IPT* genes remained nearly unchanged. This might have occurred because CKs were transported from the roots to the shoots after decapitation. The expression of the three transcripts of *ARR12* was upregulated. *ARR12* belongs to the Type-B *ARR* family and promotes *de novo* shoot regeneration in *Arabidopsis thaliana*; thus, ARR12 acts as a molecular link between CKs signaling and shoot meristem-specific genes [79,80]. The upregulation of *ARR12* expression might correlate with the upregulation of *CycD* gene expression. Sucrose has also been found to upregulate the expression of D-type cyclin genes [81]. Therefore, after plants recover from wounding, CKs might activate bud outgrowth together with sugars by upregulating the expression of cell cycle-related genes.

*DRM1* is a well-known dormancy marker gene in both herbaceous and woody plants, and its expression was repressed by sugars [82]. The expression of *DRM1* was upregulated from 0 h to 6 h but downregulated from 24 h. *DRM1* is also related to abiotic and biotic stress responses [55]. Therefore, the upregulation of *DRM1* expression might be attributed to the wound response caused by JAs, but its expression is then downregulated by sugars and CKs. The expression of *DRM1* reflected the state of dormancy of the lateral buds after decapitation. *BRC1*, an integrator of multiple signals involved in shoot branching, and its orthologs are predominantly expressed in dormant buds [23]. However, the expression levels of *BRC1* were nearly unchanged from 0 h to 48 h in this study. One possible reason for this phenomenon is that the initial growth depends on the *BRC1*-independent pathways, and *BRC1* might play a significant role after 48 h.

## 5. Conclusions

**Figure 5:**
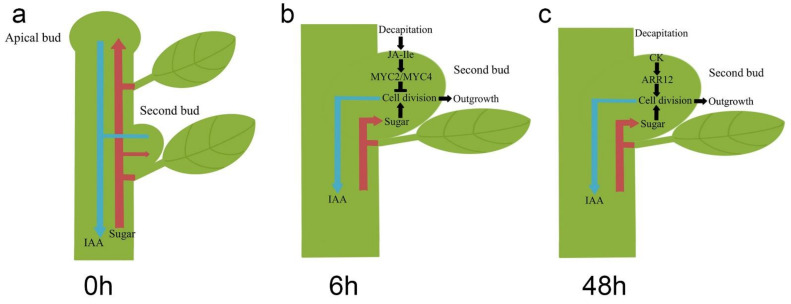
Models of second bud outgrowth after decapitation at 0 h (**a**), 6 h (**b**), and 48 h (**c**).

Internodes between the buds of chrysanthemum plants are shorter than those of other plant species, such as garden pea. Therefore, the wound response in the second buds caused by decapitation is an additional matter which should be considered in the process of bud growth in lateral buds. In conclusion, our study proposed that the second buds proceeded past the two main stages after decapitation. In intact chrysanthemum plants, lateral buds are repressed by apical buds through weaker sugar absorption and lower capacity to export IAA to PATS (Figure 5a). The apical buds of chrysanthemum plants keep growing, resulting in the increase of plant height. After decapitation, the second buds can break dormancy by exporting more IAA and absorbing more sugar. However, decapitation is also able to cause a wound response in the plants with a JA-related reaction. Therefore, in the first stage, from 0 h to 6 h after decapitation, the expression of cell cycle-related genes was inhibited, resulting in the stagnation of bud outgrowth (Figure 5b). In the second stage, from 6 h to 24 h after decapitation, the wound response caused by JA-Ile stopped, and more CKs accumulated in the second buds (Figure 5c). Both sugar and CKs promoted the expression of *CycD* genes, after which the second buds continued to develop into branches.

## Figures and Tables

**Table 1:** Statistics of NGS of each sample.

Time	BMK-ID	ReadSum	BaseSum	GC(%)	Q30(%)
0 h	T01	26,767,576	8,005,676,720	42.83	94.51
	T02	20,950,118	6,267,548,322	42.61	94.65
	T03	25,183,097	7,514,078,462	42.69	94.73
3 h	T04	22,967,608	6,873,236,908	42.94	90.51
	T06	25,210,897	7,548,853,932	42.85	91.34
	T19	28,931,725	8,613,977,646	44.06	91.09
6 h	T07	24,152,120	7,224,006,796	43.46	94.95
	T08	20,746,285	6,203,429,872	42.58	94.38
	T09	21,172,920	6,326,572,354	42.60	94.41
12 h	T10	22,846,998	6,824,592,758	42.62	94.66
	T11	22,734,631	6,794,137,036	42.45	94.47
	T12	23,235,704	6,939,240,352	42.45	94.56
24 h	T13	24,151,007	7,220,435,914	42.73	94.20
	T14	23,940,581	7,158,885,232	42.53	94.39
	T15	22,207,198	6,642,610,762	42.68	94.39
48 h	T16	23,154,529	6,913,758,850	43.25	94.54
	T17	22,872,822	6,838,511,346	42.70	94.43
	T18	23,455,701	7,000,411,940	42.81	94.59

**Table 2:** Statistics of F01 libraries of different lengths.

Library	1–2K	2–3K	3–6K	All
ROIs	113,368	105,317	78,834	297,519
Number of five prime reads	57,998	58,541	40,708	157,247
Number of three prime reads	66,054	63,609	43,561	173,224
Number of poly-A reads	63,848	62,527	43,423	169,798
Number of filtered short reads	16,314	9054	2105	27,473
Number of non-full-length reads	49,732	47,916	42,560	140,208
Number of full-length reads	47,322	48,347	34,169	129,838
Number of full-length non-chimeric reads	47,124	48,196	34,068	129,388
Average FLNC read Length	1421	2213	3619	2294
Full-length percentage (FL%)	41.74%	45.91%	43.34%	43.64%
Artificial concatemers(%)	0.42%	0.31%	0.30%	0.35%

**Table 3:** Annotation results of transcripts in F01 libraries based on different databases.

	All	GO	KEGG	KOG	Pfam	Swissprot	COG	eggNOG	nr
Annotated_Number	57,972	1789	26,357	37,374	47,880	45,307	25,052	56,606	57,629

**Table 4:** Number of DEGs in the different comparison groups.

DEG Set	DEG Number	Up-Regulator	Down-Regulator
0 h vs. 3 h	1587	869	718
0 h vs. 6 h	5247	2778	2469
0 h vs. 12 h	2785	1469	1316
0 h vs. 24 h	2773	1329	1444
0 h vs. 48 h	2351	1098	1253
3 h vs. 6 h	381	185	196
3 h vs. 12 h	570	294	276
3 h vs. 24 h	1531	680	851
3 h vs. 48 h	1427	611	816
6 h vs. 12 h	1094	451	643
6 h vs. 24 h	4169	1689	2480
6 h vs. 48 h	4377	1783	2594
12 h vs. 24 h	1176	246	930
12 h vs. 48 h	1633	563	1070
24 h vs. 48 h	600	199	401

## Data Availability

The TGS sequence data were submitted to the National Center for Biotechnology Information (NCBI; https://www.ncbi.nlm.nih.gov/, accessed on 2 April 2021) database under accession number PRJNA681921. The NGS sequence data were submitted to the NCBI database under accession number PRJNA673985.

## Supplementary Materials

The following are available online at https://www.mdpi.com/article/10.3390/biology10050440/s1, Table S1: List of predicted alternative splicing events, Table S2: List of transcripts identified as lncRNAs, Table S3: Target mRNAs predicted to interact with lncRNAs, Table S4: Functional annotation of transcripts in F01 cDNA libraries, Figure S1. Venn diagram showing the identification of lncRNAs (a) and the classification of 2,835 TFs in F01 libraries (b), Figure S2. GO annotations in the ‘0 h vs 6 h’ and ‘6 h vs 48 h’ comparisons. (a) GO annotations in the ‘0 h vs 6 h’ comparison; (b) GO annotations in the ‘6 h vs 48 h’ comparison.
