# Supplementary material for: Integrated Signals of Jasmonates, Sugars, Cytokinins and Auxin Influence the Initial Growth of the Second Buds of Chrysanthemum after Decapitation
Source: Biology (Basel). 2021 May 16;10(5):440. doi: 10.3390/biology10050440 (PMC8156878; doi:10.3390/biology10050440)
Supplement: Supplementary file 1 [file biology-10-00440-s001.zip › supplementary figures.pdf]

Supplemental Figures

Integrated signals of jasmonates, sugars, cytokinins and auxin influence the initial growth of the second buds of chrysanthemum after decapitation

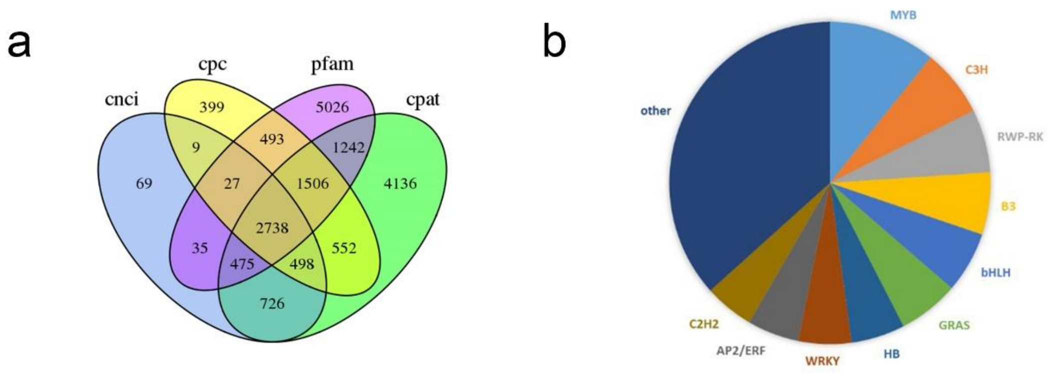

Figure S1. Venn diagram showing the identification of lncRNAs (a) and the classification of 2,835 TFs in F01 libraries (b).

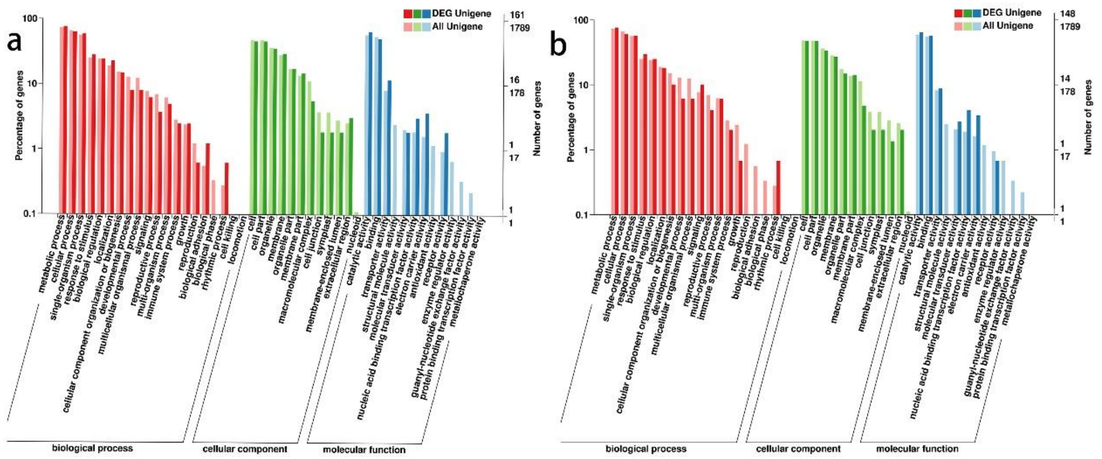

Figure S2. GO annotations in the '0 h vs 6 h' and '6 h vs 48 h' comparisons. (a) GO annotations in the '0 h vs 6 h' comparison; (b) GO annotations in the '6 h vs 48 h' comparison.
